# Valorisation of fish scales and bones: a sustainable source of bioactive proteins and collagen for nutraceuticals

**DOI:** 10.1186/s40643-025-00970-w

**Published:** 2025-12-03

**Authors:** Sivakamavalli Jeyachandran, Mohammed Aman

**Affiliations:** 1 Lab in Biotechnology and Biosignal Transduction, Department of Orthodontics, Saveetha Dental College and Hospital, Saveetha Institute of Medical and Technical Sciences (SIMATS), Saveetha University, Chennai, Tamil Nadu 600077 India; 2 Department of Industrial Engineering, College of Engineering, University of Business and Technology, 21448 Jeddah, Saudi Arabia

**Keywords:** Seafood waste valorisation, Bioactive marine peptides, Sustainable collagen sources, Enzymatic hydrolysis techniques

## Abstract

Fish scales and bones, traditionally regarded as low-value seafood by-products, represent abundant and sustainable sources of collagen and bioactive proteins with high nutraceutical potential. These tissues typically contain 30–40% organic collagen matrix and 60–70% hydroxyapatite minerals, making them rich substrates for recovery. Advances in green and enzymatic extraction methods now achieve collagen yields of up to 25–35%, with ultrasound- and microwave-assisted extraction reducing processing times from days to minutes while preserving the triple-helix structure and bioactivity. Enzymatically derived peptides demonstrate potent health-promoting effects, with antioxidant capacities comparable to or exceeding vitamins C and E, ACE-inhibitory peptides lowering blood pressure in preclinical models, and clinical trials showing that daily supplementation with 10 g fish collagen peptides for 8–12 weeks improves skin hydration, elasticity, wrinkle reduction, and reduces osteoarthritis-related joint pain. These marine biomolecules, when delivered through advanced nanocarrier systems such as nanoliposomes and nanoemulsions, exhibit enhanced bioavailability, stability, and consumer acceptability. Beyond health benefits, valorisation of fish by-products supports environmental sustainability by diverting waste and contributing to the circular bioeconomy. Despite challenges in scalability, regulatory compliance, and standardization, ongoing technological innovations position fish scales and bones as promising raw materials for next-generation nutraceuticals.

**Graphical abstract:**

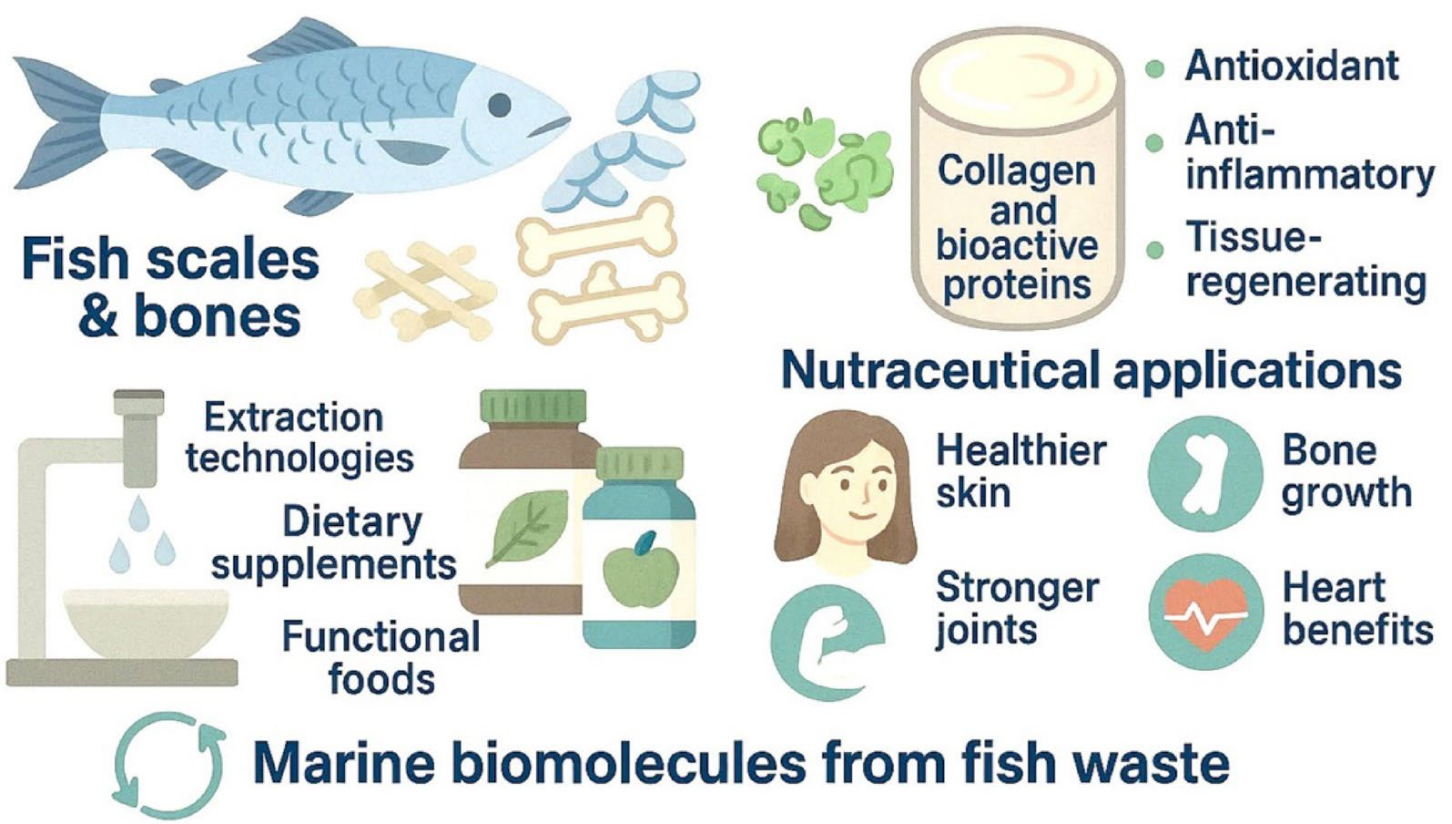

## Introduction

There is a huge amount of waste produced in the seafood industry and fish scales and bones represent much of it. Before, these were often thrown out or used for low-quality purposes which has led to both environmental damage and resource loss. On the other hand, growing awareness of sustainable methods and circular economy ideas has contributed to studies looking at using seafood by-products to produce useful biomolecules (Fraga-Corral et al. 2022; Venugopal 2022). Key attention in these biomolecules has been given to functional proteins and collagen for their widespread uses in nutraceuticals and pharmaceuticals. Vertebrates depend on collagen, their most common structural protein, for keeping skin pliable, joints flexible and bones strong. There are several facts that make fish-derived collagen, particularly from scales and bones, better than bovine and porcine collagen: it causes a lower risk of disease transmission, is biocompatible and faces fewer cultural and religious limitations. Besides this, fish collagen has important physical and chemical properties that make it ideal for use in functional foods, supplements and cosmetic products (Alves et al. 2017; Tang et al. 2022).

Along with collagen, fish scales and bones are high in proteins and peptides that provide useful benefits such as antioxidant, anti-inflammatory, antimicrobial and antihypertensive effects (Xu et al. 2023; Salindeho et al. 2022). Such bioactive compounds may be very important for human health since they can help protect against inflammation, reduce stress on the heart, lower oxidation and support overall longevity (Zaky et al. 2022; Nasri 2019). For decades, fueling the process of extracting and purifying functional proteins and collagen from fish by-products has depended on using chemicals such as acids and bases. Effective extraction methods including enzymatic hydrolysis, ultrasound-assisted extraction and supercritical fluid extraction are eco-friendly and far more emerging technologies to come in future (Ramakrishnan et al. 2023; Gaikwad and Kim 2024). By improving both the amount and quality of extracted biomolecules, they also help the seafood industry meet its eco-friendly goals by generating less waste and consuming less energy. While scientists continue to uncover new applications for fish by-products, several challenges remain, including variability in their biochemical composition, fluctuations in the levels of desired compounds, and the pressing need for comprehensive validation through clinical trials (Nawaz et al. 2020). In addition, expanding extraction methods to bigger scales and complying with laws is still a big challenge for large-scale manufacturers. The many uses of fish scales and bones, along with rising interest in natural and sustainable nutraceutical products, have made it necessary to review key studies, problems and future trends in this area. This review evaluates the biochemical structure, procedures for getting these biomolecules, how they act and how they might be explored and used in nutraceuticals and for better seafood industry practices, supporting human health.

## Composition of fish scales and bones and its importance for valorization

**Fig. 1:**
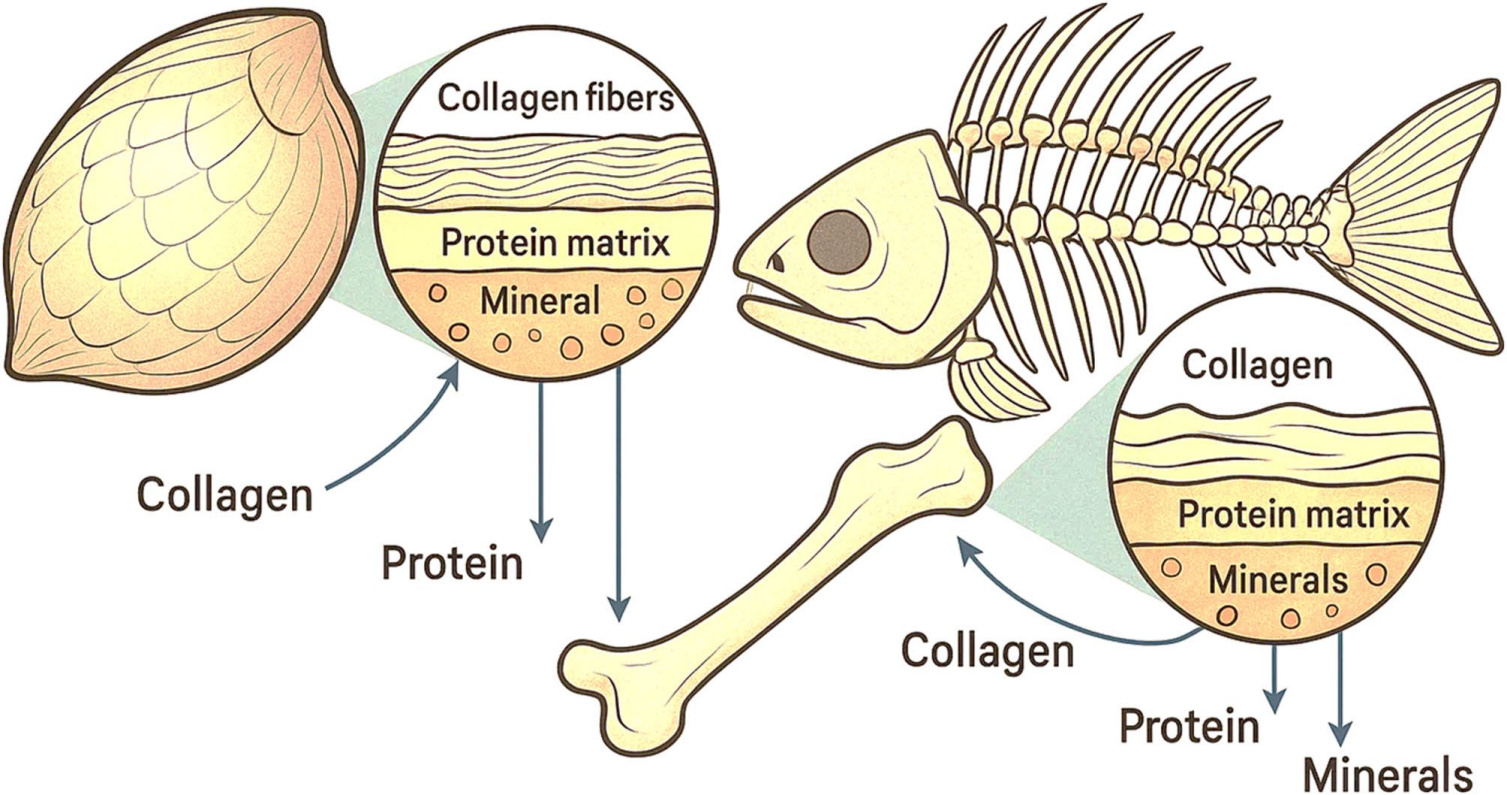
Structure and composition of fish scales and bones

With raising demand and its interest in utilizing sustainable products increasing, the seafood industry now recognizes that fish scales and bones are the rich sources of collagen and other beneficial bioactive compounds including proteins and peptides. In the past, these materials are considered as waste/debris whereas in the present with notable scientific advancements these fish scales and bones are found to be reservoir of bioactive compounds and its applications are benefitted in different fields such as biomedicine, cosmetics and nutraceuticals. (Table 1) (Fig. 1) (Coppola et al. 2021; Qin et al. 2022).

**Table 1:** Bioactive peptides derived from marine sources: bioactivity, molecular weight, physicochemical properties, and biocompatibility

Bioactive peptide	Marine source	Bioactivity	Molecular weight (Da)	Physicochemical properties	Biocompatibility & biodegradability	References
Collagen peptides	Fish scales (Cod, Salmon)	Antioxidant, Anti-inflammatory	500–3000	High solubility, gelation, emulsifying properties	High biocompatibility, biodegradable, low immunogenicity	Tawalbeh et al (2025)
ACE-inhibitory peptides	Fish bones (Tilapia)	Anti-hypertensive	500–1500	Stable under physiological pH, hydrophobic amino acid residues	Biodegradable, non-toxic, compatible with human enzymes	Wang et al. (2024); Kendler et al. (2024)
Antioxidant peptides	Fish collagen hydrolysate	Radical scavenging, Metal chelation	400–2500	Good water solubility, stable secondary structure	Biocompatible, biodegradable with minimal side effects	Cadar et al. (2024)
Anti-inflammatory peptides	Fish skin (Atlantic salmon)	Inhibition of pro-inflammatory cytokines	600–2000	Amphiphilic nature, good emulsification	High compatibility, biodegradable, promotes cellular uptake	Blumhardt (2015)
Immunomodulatory peptides	Marine shrimp	Immune system enhancement	800–2200	Moderate solubility, stable in physiological conditions	Excellent biocompatibility, biodegradable, safe in vivo	Kumar et al. (2023)
Antimicrobial peptides	Marine mollusks (Abalone)	Antibacterial, Antifungal	1000–3000	Cationic, amphipathic, interacts with microbial membranes	Biocompatible, rapid biodegradation, minimal cytotoxicity	Rodrigues et al. (2025); Wu et al. (2021)
Neuroprotective Peptides	Fish muscle proteins	Neuroprotection	500–2000	Hydrophilic, resistant to enzymatic degradation	High biocompatibility, crosses blood–brain barrier potential	Abuine et al. (2019)
DPP-IV inhibitory peptides	Tilapia skin gelatin and collagen	Antidiabetic	400–1500	Small size, hydrophobic amino acids	Biodegradable, non-toxic, compatible with gut enzymes	Liu et al. (2022)
Anti-cancer Peptides	Marine sponges, tunicates and algae	Cytotoxicity against cancer cells	< 1000	Amphiphilic, stable tertiary structure	Selectively cytotoxic, biodegradable, low side effects	Zhang et al. (2021)
Wound healing peptides	Fish scale collagen peptides/ chito-oligosaccharides	Promotion of tissue regeneration	700–2500	Excellent film-forming, good water retention	Biocompatible, biodegradable, promotes cell adhesion	Wang et al. (2011)
Calcium-Binding Peptides	Alaska Pollak, Fish bone hydrolysates	Bone mineralization and health	800–3000	High affinity to calcium ions, stable under physiological pH	Biodegradable, enhances bone cell compatibility	Pérez et al. (2024)
Hypo-cholesterol peptides	Tuna skin collagen, preadipocytes	Cholesterol-lowering	600–2000	Amphiphilic, moderate solubility	High biocompatibility, biodegradable, safe for oral use	Liu et al. (2023)
Anti-fatigue peptides	Fish muscle protein hydrolysates (*Pseudosciaena crocea*)	Improvement in endurance, fatigue reduction	800–2500	Good solubility, stable under moderate heat	Biodegradable, compatible with human metabolism	Zhao et al. (2016)
Anti-diabetic peptides	Fish scale collagen peptides	Regulation of blood glucose and insulin sensitivity	400–1800	Hydrophobic residues enhance bioactivity	Biocompatible, enzymatically digestible	Balakrishnan et al. (2022)
Anti-obesity peptides	Marine fish protein hydrolysate	Fat metabolism regulation	500–2200	Amphiphilic, soluble in aqueous solutions	Biodegradable, no cytotoxicity reported	Manikkam et al. (2016)
Antiviral peptides	Marine algae and fish by-products	Inhibition of viral replication	1000–3500	Cationic, binds viral envelopes	Biocompatible, biodegradable, safe for therapeutic use	Laroche et al. (2018)
Anti-osteoporotic peptides	Fish bone-derived peptides	Promotion of bone density and strength	900–3200	Strong calcium-binding affinity	Biodegradable, supports osteoblast activity	Zhang et al. (2024a)
Metal-chelating peptides	Fish scale protein hydrolysates	Heavy metal detoxification	500–2000	High affinity for metal ions	Biocompatible, enhances detox pathways	Yu et al. (2024)
Anti-thrombotic peptides	Marine fish protein hydrolysates	Prevention of blood clot formation	700–2500	Hydrophilic, anticoagulant activity	Biodegradable, safe anticoagulant alternative	Dwivedi and Pomin (2020)
Anti-hyperglycemic peptides	Fish muscle hydrolysates	Blood sugar regulation	400–1600	Small size, high stability in digestive tract	Biocompatible, effective in glucose metabolism regulation	Sharkey et al. (2020)
Anti-melanogenic peptides	Chitosan nanoparticles	Skin whitening and pigmentation control	600–2000	Amphiphilic, stable under UV exposure	Biodegradable, low toxicity in dermal applications	Hatem et al. (2022)
Anti-hypertensive peptides	Fish muscle collagen peptides	Blood pressure regulation	400–1500	Stable secondary structure, hydrophobic amino acid content	Biodegradable, minimal side effects	Abachi et al. (2019)

Fish scales consist mostly of collagen and, more than half of that collagen, is type I collagen. Scales are strengthened and can flex by this collagen. Rigidity and defense in fish come from the presence of hydroxyapatite in their inorganic part (Dhara et al. 2013). Magnesium and carbonate trace minerals are found and contribute to the structure of the scales (Lall and Kaushik 2021). Most of the dry weight in fish bones comes from a mineralized collagen matrix which they have in common. There are roughly 30–40% of the organic matrix is made up of collagen types and other proteins, with a main mineral fraction of hydroxyapatite at 60–70%. This brought-together material makes bones tough and flexible for supporting the body. Besides collagen, both fish scales and bones have bioactive peptides and proteins that fight oxidation, treat infection, inflammation and high blood pressure (Salindeho et al. 2022) which gives them significance in health applications. Boiling collagen and proteins out of fish scales and bones is made difficult by their complex hierarchy. Each scale consists of an outer layer full of minerals and an inner protein layer packed tightly with collagen in a triple helix (Ottani et al. 2002). Adding minerals makes the tissue stronger, yet collagen needs acid or enzyme treatment to loosen and dissolve them so the tissue can be extracted (Matinong et al. 2022). Just like the rest of bone, fish bones have collagen phasing through a mineralized hydroxyapatite matrix. Having collagen and mineral phases crosslinked requires special protease or strong acid treatment to free the collagen and help the release of functional peptides (Rýglová et al. 2017). Presence of proteoglycans and types of non-collagenous proteins in the extracellular matrix affect both the amount and top-quality of extracted collagen. Recognizing these structural aspects enables one to choose the best pH, temperature, type of enzyme and processing time for maximum recovery and bioactive protection of collagen and peptides. Fish scales and bones look different from one species to another and this affects how much you can get and how pure the biomolecules will be. On average, cod and salmon contain more collagen and different amino acids in their muscles than catfish does (Tylingo et al. 2016). Heavily scaled and larger species need stronger methods to process and remove minerals. Other things that matter include the environment, diet, age and season of the year. Collagen is more extractable from younger fish because it is less tied together in their flesh, whereas the collagen networks in older fish become linking, reducing its ability to be absorbed but improving its strength. Knowing that different organisms differ greatly enables us to develop standard extraction techniques and produce biomolecules with reliable properties and functions for industry.

Utilization of seafood by-products, including fish scales and bones, is of great value as a source of biomolecules for broadly useful applications and as a means to valorize these by-products and leverage their inherent worth as a sustainable source of value. Fish scales and bones which had traditionally been seen as waste, contribute sizeable to the residual biomass produced by the seafood processing industry. From the beginning, disposal of these fish waste materials has, been of serious environmental concern because of harmful pollution, greenhouse gas emissions and wastage of resources. While it is well known for its by-products, everyday these by-products are seen as renewable and sustainable resources for high value biomolecules such as collagen, functional protein, peptides and minerals (Cao et al. 2022; Darda et al. 2021). Most of fish scales are of collagen, a structural protein which needs to be present in the skin for elasticity, joints for flexibility and bones for health. Because of its biocompatibility and low risk of disease transmission, collagen derived from fish scales is highly attractive in biomedical, cosmetic and nutraceutical applications, compared to terrestrial sources (Farooq et al. 2024). Likewise, fish bones are replete with calcium phosphate and collagen, minerals and proteins with added strength to bone and are therapeutic when extracted and processed properly. Being of mainly biochemical composition, fish bones are a good raw material for the manufacturing of functional foods and dietary supplements for supporting musculoskeletal health (Granito et al. 2018; Benjakul et al. 2019). In addition to collagen, fish scales and bones are a source of bioactive peptides and proteins posing antioxidant, anti-inflammatory, antimicrobial and antihypertensive activities. Recently, due to their potential as prevention against disease, these bioactive compounds have stimulated much attention as to their activities in reducing oxidative stress and inflammation, both hallmarks in chronic conditions for example cardiovascular diseases and aging related disorders (Ashraf et al. 2020; Chen et al. 2021). Recovery of these molecules from fish by product provides a sustainable source of additives for feeding in line with a growing consumer demand for natural and health promoting ingredients. Fish scales and bones valorizations also strongly match with circular economy and sustainability principles. Converting iterarily considered waste into valuable biomolecules mitigates environmental pollution, reduces exposure of pollutions to the basins and minimizes land filling, hence mitigating the negative ecological footprint of seafood processing. Through closing the loop within the seafood supply chain, it lowers demand of non-renewable raw materials (Maschmeyer et al. 2020). Valorization is economically important in that it supplies new revenue streams to the seafood industry by turning low value by products into high demand nutraceuticals, cosmetics and biomedical materials and in doing so enhances the profitability and innovation in the seafood industry. The feasibility and efficiency of recovering multiple biomolecules from fish scales and bones with preserved functional properties have been improved by recent advances in extraction and purification technologies such as enzymatic hydrolysis and the use of green extraction methods (Caruso et al. 2020). These technologies facilitate the production of high-quality collagen and bioactive peptides that can be used in a very broad spectrum of consumer product applications such as dietary supplements and skin care formulations.

Human health and some more physiological processes, depend on essential roles being played by important biomolecules such as functional proteins and collagen. Proteins with specific activity that extends beyond their nutritional value (enzymatic, antioxidant, antimicrobial or immunomodulatory), are called functional proteins (Pedro et al. 2022). These proteins, generally obtained from natural sources such as seafood byproducts, help improve health via their control of oxidative stress, cell signaling and inflammation, the pathways that prevent and maintain health (Kemp and Kwon 2021). Collagen, the primary constituent of skin, bone, cartilage, tendons and ligaments, is an insoluble structural protein that makes up about 30 per cent of all protein in the human body (Balasubramanian et al. 2013). Tensile strength, elasticity and structural integrity is given to tissues. Collagen is important in several human health processes such as wound healing, skin hydration, joint flexibility and bone regeneration (Chen et al. 2021). The age-related degradation of collagen is associated with common degenerative conditions such as osteoarthritis, skin aging and osteoporosis, so there has been an increased interest in collagen supplementation (Wang 2021). Collagen from marine source, mainly fish scales and the bones, has specific advantages including higher bioavailability, lower allergenicity and fewer religious and cultural restrictions, when compared with mammalian collagen (Islam and Mis Solval 2025). These sources provide promising bioactivities of functional protein and collagen, including antioxidant, anti-inflammatory and antihypertensive properties which makes them interesting candidates for nutraceutical development. In this present review, fish scales and bones as a sustainable and rich source of functional proteins and collagen will be comprehensively evaluated and their possible applications to nutraceutical industry will be highlighted. Increasingly, consumers seek natural and preventive health solutions and nutraceuticals (food derived products that provide health benefits beyond basic nutrition) are whole food commodities providing such benefits (Joy et al. 2024). The extraction methods, functional properties, health benefits and potential for commercialization of seafood byproducts with a focus on valorization of seafood byproducts to address both health and environmental sustainability challenges are discussed. Reasons for understanding nutraceutical aspects of fish derived proteins and collagen are: First, it supports principles of circular bioeconomy in the seafood industry and promotes resource efficiency and waste reduction. Second, it helps to create functional foods and supplements geared to enhancing cardiovascular health, skin aging, joint function and overall health. It finally encourages innovation in sustainable health product development in line with increasingly common natural and green health interventions in the world.

## Overview of seafood by-products

By fish and other marine organisms shipped overseas, seafood by product is the parts of fish and other marine organisms left after processing the primary edible portions for human consumption. Fish scales, bones, skin, fins, head, viscera (internal organs), shells and other residual tissues composed the bulk of the waste generated by the seafood processing industry which is represented as these by products (Coppola et al. 2021). Traditionally considered as waste or used for low value applications like animal feed and fertilizers, these by products are now understood to be sources of valuable raw materials containing bioactive compounds, proteins, lipids, minerals and other nutrients (Jayathilakan et al. 2012). One of the major by product as filed under fish scales is made up of almost purely collagen and hydroxyapatite, thus causing fish scales a good source of functional proteins and biominerals (Qin et al. 2022). On the other side, fish bones are rich in calcium phosphate, collagen, etc., substances that contribute to the possible strengthening of bone and joints by proper procession (Pérez et al. 2024). Type I collagen in fish skin has drawn much attention in nutraceuticals, cosmetics and biomedical material (Rajabimashhadi et al. 2023). Fish heads and viscera and shells of crustaceans (shrimp, crab), also hold important collections of oils that are rich in omega 3 fatty acids and enzymes and chitin and chitosan compounds with many uses in the food and pharmaceutical industries (Abuzar 2023). While knowing about their potential value is increasing, much seafood by product remains thrown away or handled poorly. However, choices to landfill, dumping in water bodies, incineration or converting into products with low value such as animal food or fertilizer (Singh et al. 2022) dominate its disposal. Often, they are inefficient and they present major environmental and economic concerns. By far, both landfilling and ocean dumping caused soil contamination, water pollution and greenhouse gas emissions (due to organic waste decomposition) (Siddiqua et al. 2022). Moreover, improper management of seafood waste attracts pests and produces bad odours which badly affect the local population surrounding seafood processing plants (Islam et al. 2004). Additionally, there is a significant opportunity to realize both economic and sustainable utilization losses of valuable bioactive compounds that are discarded.

However, the environmental impact of how seafood by products is disposed continues to be understood as an important problem. Fast decomposition of organic seafood processing waste releases methane and other greenhouse gases which contribute to climate change (Venugopal and Kim 2025). Eutrophication resulting from discharge of untreated seafood waste into aquatic systems has caused oxygen depletion and therefore adverse effects on aquatic biodiversity (Jan et al. 2022). In addition, pathogens, heavy metals and other contaminants present risks to environmental and human health if seafood by product is not properly treated (Mol and Coşansu xxxx). This pile up of such waste can harm fisheries, tourism and local economies that rely on clean marine environments. Since these challenges exist, a number of international and national policies have focused on waste minimization, recycling and resource recovery in the seafood industry (Ginikanwa et al. 2024). In seafood processing, biorefinery concepts and circular bioeconomy models are being developed to drive down generation of waste by converting by-products into high values compounds, thereby lowering environmental perturbation as well as stimulating economic development (Sarangi et al. 2023). Over recent years there have been an increased focus on green technology and biotechnological method for extraction of valuable compounds from seafood by products sustainably (e.g. enzymatic hydrolysis, ultrasound assisted extraction) (Venugopal et al. 2023). In recent years, efforts have been made throughout the globe to set up integrated processing systems that combine waste management with product development (Ganjeh et al. 2023). While these advances solve some problems, complications remain in standardizing extraction methods, enhancing product safety and crafting processes that will be commercially viable. These issues are foundational for converting seafood by-products from environmental liabilities to stable, sustainable resources with economic value.

## Extraction, purification, and characterization of collagen and functional proteins from fish bones and scales

Functional proteins and collagen must be extracted and purified from fish scales and bones to create valuable biomolecules for nutraceuticals, pharmaceuticals and cosmetics. Appropriate approaches to extracting collagen are engineered to get the best yield and maintain its 3D shape which affects their usefulness and usefulness for therapy (Dong and Lv 2016). Many experiments on collagen recovery have depended on traditional extraction techniques. When extracting acid-soluble collagen, acetic, lactic or citric acids are usually used to softly dissolve the mineral particles and release collagen (Farooq et al. 2024). Treatments made with sodium hydroxide or sodium carbonate are commonly used to break down non-collagenous proteins and other impurities. Still, using these chemicals can lead to damage in the way collagen functions and works. Because these old processes are both cost-saving and easy to use, they can cause the products to be less safe and can produce hazardous waste. Meanwhile, Enzymatic Hydrolysis is now seen as a safer and more environmentally-friendly approach. The pepsin, trypsin, papain and alcalase among the proteolytic enzymes, collagen’s non-helical telopeptide parts are cleaved and made soluble which helps improve the collagen yield for extraction, preserving the helix structure of the collagen (Yang et al. 2024). Using enzymes to break down collagen enhances its properties, so that it becomes active against damage from free radicals, hypertension and inflammation. With this method, green chemistry principles are followed by using less chemistry and generating less waste. Having such systems used on a larger scale is held back by the high cost of enzymes and finding optimal settings for hydrolysis. Subsequent to recent innovations in UAE, MAE and SFE, fish by-products are now more efficiently used for collagen and protein extraction, taking less time and preserving the functional characteristics of the proteins (Al Khawli et al. 2019). The UAE uses powerful sound vibrations to break the collagen-mineral links in raw material which makes it easier for solvents to enter and leads to better output and faster processing. In a similar way, MAE speeds up heating of the solvent and matrix using microwaves which leads to faster collagen solubilization and less energy use (Zhang et al. 2024b). Because supercritical extraction involves no toxic solvents, more people are selecting it to create clean and high-quality collagen for pharmaceutical and food applications (Singh et al. 2022). This technology faces some issues that must be improved before it can balance costs and be scaled up. Extracted collagen must undergo purification to remove any contaminants, salts and small molecules. Removal of small contaminants in electronics is possible with dialysis through semipermeable filters that help enhance purity. It is possible to concentrate and separate collagen peptides by size and formulate them according to need using ultrafiltration and membrane systems. To refine and separate particular active parts from collagen and protein, several chromatographic approaches including ion-exchange, gel and affinity are applied (Dancila and Bosomoiu 2024). For nutraceutical and biomedical use, purifying the product is vital because it ensures it is safe, effective and follows all required rules (Table 2).

**Table 2:** Comparison of conventional and advanced extraction techniques for fish collagen and proteins

Method	Yield (%)	Purity	Molecular integrity	Processing time	Cost	Environmental impact
Acid-soluble collagen (ASC)	10–15%	Moderate; residual non-collagen proteins	Triple-helix partly preserved; risk of denaturation	Long (24–72 h)	Low	High chemical use; acidic waste generation
Pepsin-soluble collagen (PSC)	20–30%	High; removal of non-collagenous proteins	Triple-helix well preserved	Moderate (12–48 h)	Moderate (enzyme cost)	Reduced chemical waste vs. ASC; still moderate environmental load
Ultrasound-assisted extraction (UAE)	25–35%	High	Maintains triple-helix and bioactivity	Short (30–120 min)	Moderate	Eco-friendly; reduced solvents and energy use
Microwave-assisted extraction (MAE)	25–35%	High	Good preservation; risk of overexposure damage	Very short (15–60 min)	Moderate to High (equipment cost)	Low solvent use; energy-efficient
Supercritical fluid extraction (SFE)	20–30%	Very high	Excellent preservation of molecular integrity	Short to Moderate (1–3 h)	High	Green method; non-toxic solvents (e.g., CO₂)

To evaluate the quality and integrity of extracted collagen and proteins, several analytical techniques are commonly applied. SDS-PAGE provides information on molecular weight distribution and purity by separating proteins into distinct bands; intact type I collagen typically appears as α- and β-chains, and the absence of additional bands indicates minimal contamination or degradation. FTIR analysis reveals characteristic amide I, II, and III bands, where a strong amide I peak near 1650 cm⁻^1^ confirms the presence of the collagen triple helix, and shifts or reductions in these bands suggest structural alterations. CD spectroscopy is used to assess the preservation of secondary structure, particularly the polyproline II helix; a positive peak near 220 nm and a negative peak around 197 nm indicate intact triple-helical conformation, while changes in ellipticity denote partial unfolding. DSC measures the denaturation temperature (Td), which reflects collagen’s thermal stability; values in the range of 35–45 °C are expected for fish collagen, and higher Td indicates improved molecular integrity and suitability for applications requiring stability. Together, these techniques not only confirm purity and structural preservation but also provide insight into the functional performance and potential shelf life of marine-derived collagen and peptides (Jafari et al. 2020). These methods direct the improvement of extraction and purification procedures to enhance the bioactivity of the compounds. Advances have been achieved, but the process of scaling up allows industrial plant use is still a challenge. Influences on commercial processes include issues such as how much raw materials change; cost levels; their effect on the environment; and getting authorization from regulators (Liu et al. 2022). At present, the goal is to apply green methods and follow them with downstream purification to create better, greener and more economical production of marine collagen and functional proteins.

Conventional methods such as acid hydrolysis, enzymatic hydrolysis, and alkaline processing have long been fundamental for extracting collagen and functional proteins from fish scales and bones because they are simple, cost-effective, and efficient. Among them, acetic acid hydrolysis is the most widely used technique for isolating collagen from mineralized fish by-products. Natural acids like acetic, citric, or lactic acid soften collagen by dissolving the bonds between collagen fibrils and hydroxyapatite, the principal mineral matrix (Qin et al. 2022). This process yields acid-soluble collagen (ASC) that generally retains its triple helix structure, essential for its bioactivity. Mild acid treatment helps relax collagen fibers, separating them from other proteins and minerals, enabling efficient recovery (Ottani et al. 2002). However, acid hydrolysis is time-consuming, and if not carefully controlled, can lead to collagen denaturation, compromising its structural integrity and biological activity. Enzymatic hydrolysis has gained preference for being environmentally friendly and more selective. Proteolytic enzymes such as pepsin, trypsin, papain, and alcalase cleave the telopeptide regions of collagen, enhancing solubility and yielding pepsin-soluble collagen (PSC) with improved bioactivity, including antioxidant and antihypertensive properties (Harris et al. 2021). These mild conditions preserve collagen integrity and minimize chemical pollution (Cadar et al. 2024), although high enzyme costs and the need for precise control of temperature, concentration, and pH limit scalability (Rajabimashhadi et al. 2023). Alkaline treatments, using sodium hydroxide or calcium hydroxide, are also employed to remove non-collagenous proteins, fats, and minerals either before or after acid or enzyme treatment (Limeneh et al. 2022). When used in combination with hydrolysis or enzymatic extraction, these multistep processes can enhance collagen purity and yield (Matinong et al. 2022; Daboor et al. 2010). Despite their effectiveness, conventional extraction methods are slow, can damage collagen, and pose environmental risks due to chemical usage, prompting the integration of green technologies like ultrasound- and microwave-assisted extraction for faster, cleaner, and more efficient collagen recovery.

**Fig. 2:**
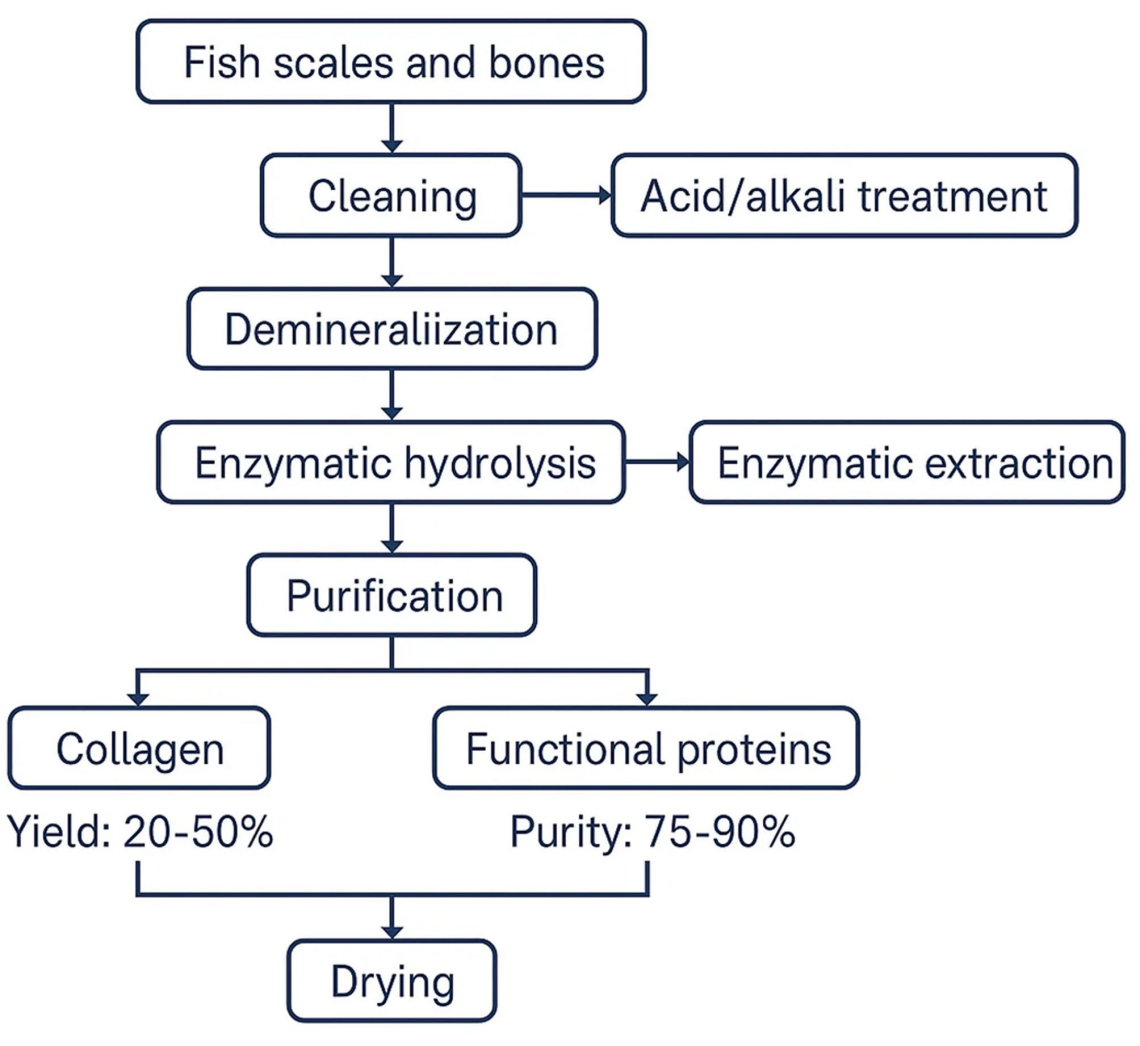
Extraction process of collagen and functional proteins from fish scales and bones

In response to the growing demand for sustainable and eco-friendly bioproducts, advanced extraction methods have been developed to improve yield, efficiency, and product quality while minimizing solvent use and processing time (Fig. 2). Ultrasound-assisted extraction (UAE) utilizes high-frequency sound waves to create microbubbles that collapse, disrupting tissues and facilitating solvent penetration. This mechanical effect accelerates collagen extraction, improving yield and maintaining the triple helix and bioactivity of collagen, while also consuming less energy and requiring fewer hazardous chemicals (Zhang et al. 2024b). Microwave-assisted extraction (MAE), on the other hand, evenly heats the solvent and raw material through molecular vibration, breaking hydrogen bonds between collagen and minerals. MAE has demonstrated faster processing times, higher collagen yields, and improved thermal stability compared to conventional acid or enzymatic methods (Mohamad et al. 2023). However, excessive microwave power or duration can damage collagen and reduce its activity, underscoring the need for careful optimization. These green methods are often combined with enzymatic extraction, where enzymes like pepsin, papain, alcalase, and trypsin selectively hydrolyze amino acid sequences to produce collagen peptides with high bioactivity (Tawalbeh et al 2025). Innovations such as immobilized enzymes and synergistic ultrasound-enzyme systems have enhanced enzyme reusability and collagen recovery efficiency (Xu et al. 2023; Amirrah et al. 2022). Although enzyme production costs remain a limitation, these advancements have significantly contributed to more sustainable, scalable, and environmentally responsible collagen extraction processes, aligning with industry and regulatory goals for green marine bioproducts.

**Fig. 3:**
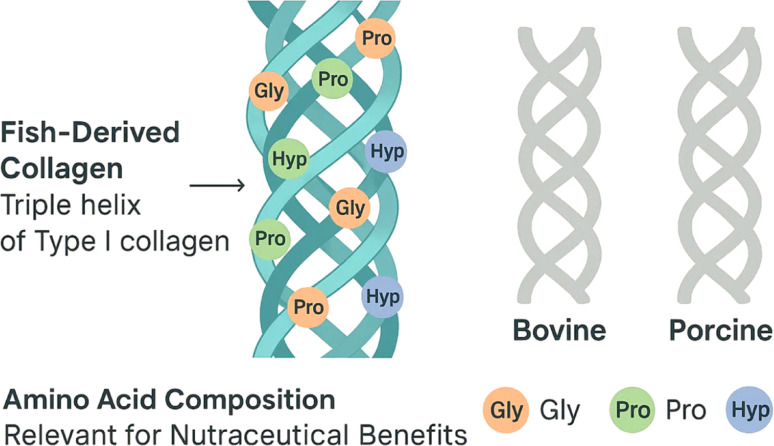
Molecular structure of fish-derived collagen

The efficiency and quality of collagen extraction depend on multiple parameters, including temperature, pH, extraction duration, and the enzyme type employed. Temperature strongly influences both solubility and molecular stability; moderate heating between 20 and 40 °C enhances extraction rates without disrupting the collagen’s triple helix, preserving its antioxidant activity and biofunctionality (Fig. 3) (Kameshwar Sharma et al. 2022). Acidic conditions (pH 2–5) effectively solubilize collagen by disrupting ionic bonds with mineral components, while enzymes exhibit optimal activity within specific pH ranges—pepsin in highly acidic conditions (~ pH 2) and alcalase in neutral to slightly alkaline environments (Rajabimashhadi et al. 2023). Deviations from optimal pH can alter enzyme activity and peptide profiles, leading to inconsistent results. Extraction duration also affects yield and quality: prolonged processing can increase yield but cause peptide degradation, while shorter, optimized treatments maintain bioactivity and structural integrity (Ahmad et al. 2017). Techniques like ultrasound or microwave-assisted extraction drastically reduce processing time from days to minutes, preserving collagen’s molecular weight distribution and biological functions (Khadhraoui et al. 2021). The choice of enzyme determines the size and activity of resulting peptides; pepsin maintains the triple helix by removing telopeptides, while alcalase generates smaller, more bioactive peptides (Ashaolu et al. 2024; Pulikkottil 2024). Current research emphasizes enzyme combinations and immobilization strategies to improve yield and tailor collagen properties for specific applications.

Once collagen and functional proteins are extracted, purification and characterization ensure their safety, bioavailability, and suitability for biomedical, nutraceutical, and cosmetic uses. Dialysis is typically employed first to remove salts, acids, and low molecular weight impurities, concentrating the collagen solution. Membrane filtration further separates proteins by molecular size, allowing fractionation of bioactive components (Bunyaratavej and Wang 2001). Advanced purification methods such as ion-exchange and size-exclusion chromatography efficiently separate proteins by charge and molecular dimensions, while affinity chromatography, though less common, is increasingly valued for its selectivity in isolating functional peptides (Chakraborty et al. 2022). Purity and molecular weight distribution are routinely assessed using SDS-PAGE, which evaluates extraction and purification efficiency (Matsumoto et al. 2019). Structural and functional integrity are confirmed using FTIR and CD spectroscopy; characteristic amide bands in FTIR signify intact collagen, while CD spectra verify proper folding and helical stability. Differential scanning calorimetry (DSC) measures denaturation temperature, indicating thermal stability and overall quality (Rochdi et al. 2000). Improved thermal stability correlates with enhanced product longevity and functional performance in formulations. Collectively, integrating multiple purification and analytical techniques provides a comprehensive understanding of collagen quality and supports its effective application in pharmaceuticals, cosmetics, and functional foods.

## Properties of extracted proteins and collagen

### Bioactive peptides and their health benefits

**Fig. 4:**
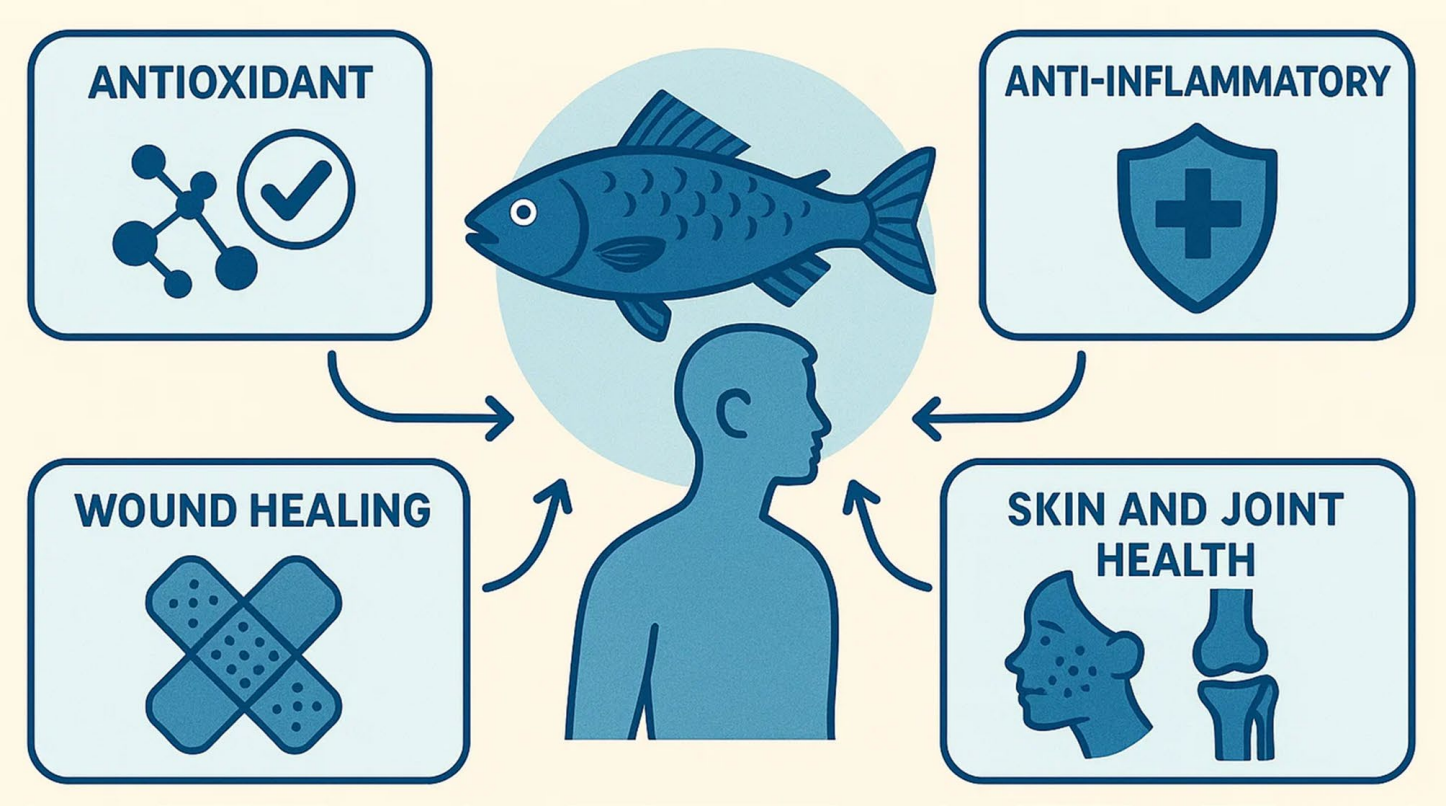
Functional properties and nutraceutical benefits of fish-derived proteins and collagen

**Fig. 5:**
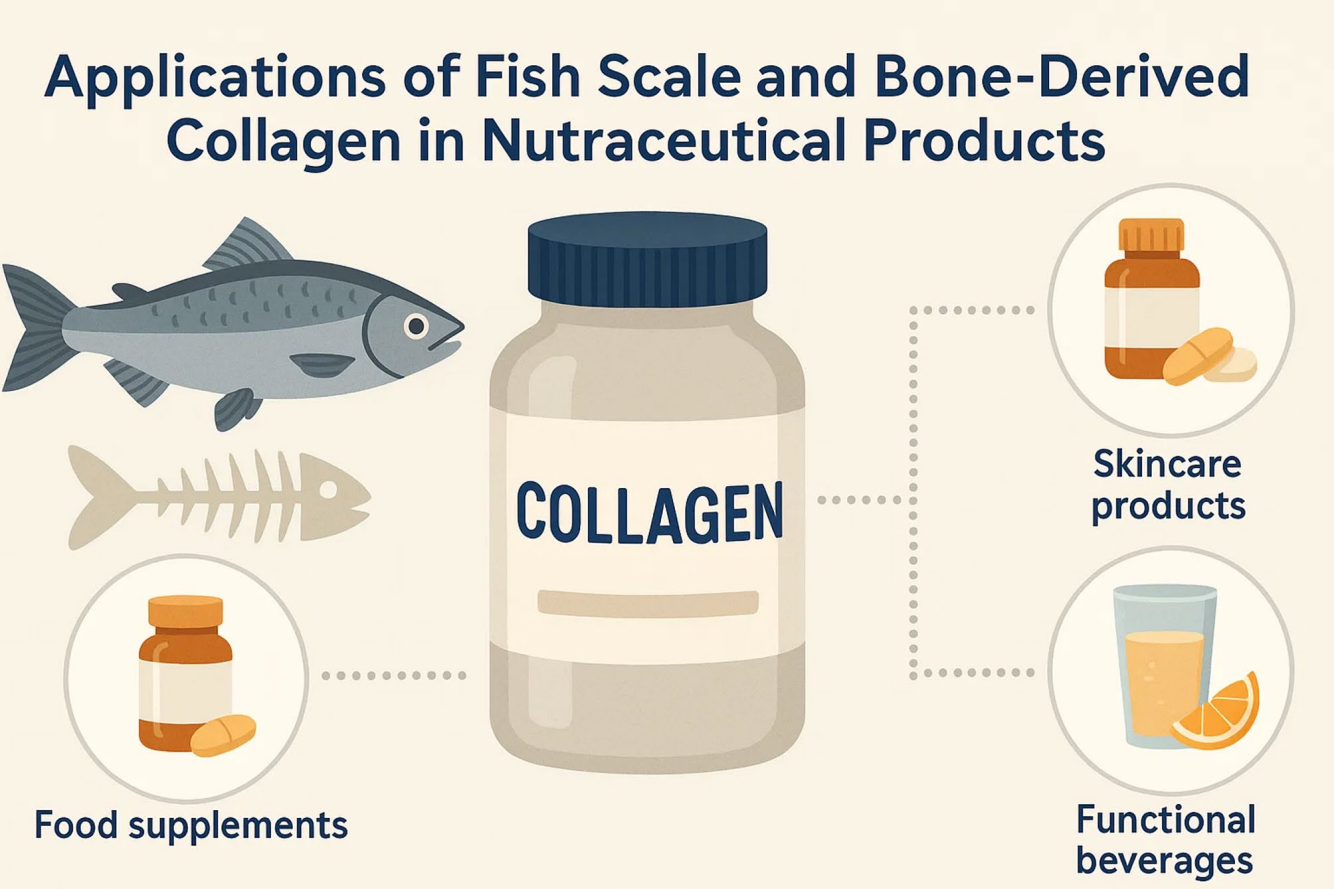
Applications of fish scale and bone-derived collagen in nutraceutical products

Lately, bioactive peptides from fish scales, bones and other seafood residues have attracted attention because of their many potential therapeutic uses (Fig. 4) (Table 3). In addition to nutrients, these specific protein pieces benefit our bodies and may even protect against or control chronic diseases. Some of the main effects of marine-based bioactive peptides are their antioxidant, anti-inflammatory and antihypertensive properties, joined together to help prevent aging and treat chronic diseases of the heart and immune system, like those affecting the cardiovascular system and cancer (Shahidi and Saeid 2025). The antioxidant activity of bioactive peptides from fish collagen and proteins was confirmed by their ability to keep cells safe from damage caused by free radicals (Fernando et al. 2023). The antioxidant properties of these peptides depend on removing harmful radicals, taking away ionic particles and preventing lipid peroxidation. Recently, investigations have pointed out that peptides made by enzymatically breaking down fish collagen are strong antioxidants, either as powerful or even better than vitamins C and E (Najafian and Babji 2012). Research has proved that hydrolysates extracted from fish scales and bone can block oxidation in the test tube, as well as in an animal’s system, improving the activities of antioxidant enzymes (Shen et al. 2019). Non-communicable diseases such as arthritis, diabetes and heart problems are frequently caused by ongoing inflammation. Some marine-derived peptides have been found to fight inflammation by altering important pathways and the release of inflammatory cytokines (Kang et al. 2019). Fish collagen hydrolysate absorbable peptides are able to suppress the secretion of TNF-α, IL-6 and COX-2, supporting lower inflammation in body tissues and cells. According to the researchers, taking collagen peptides can improve the symptoms and movements of arthritis patients and improve their daily living (Zdzieblik et al. 2017). Because of their anti-inflammatory functions, they may replace conventional anti-inflammatory medicines which are widely known to produce unpleasant side effects. Researchers have found that bioactive peptides extracted from fish by-products have potential as ACE inhibitors which help reduce blood pressure because they stop angiotensin I from being converted into angiotensin II (Pujiastuti et al. 2019). Such peptides from fish scale and bone hydrolysates show strong ACE inhibition and help reduce blood pressure in animals. Much of these peptides have short sequences in their amino acid chain filled with hydrophobic and positive side chains which improves their binding to ACE, helping them inhibit enzyme activity. Certain peptides function as ACE inhibitors and antioxidants-plus anti-inflammatory agents, leading to a stronger cardiovascular defense (He et al. 2013). Also, because they are safe and naturally produced, these peptides appear promising for use as nutraceuticals against hypertension and progression of heart disease. In recent studies on fish collagen peptides, participants with high blood pressure showed better blood pressure results and healthier vessels (Fig. 5) (Koizumi et al. 2018).

**Table 3:** Functional Protein Ingredients Derived from Marine Sources: Functional Properties, Applications, and Relevant References

Functional protein ingredient	Marine source (fish species)	Functional properties	Typical applications	References
Hydrolyzed collagen peptides	Fish scales and skin (Cod, Salmon, Tilapia, Atlantic Salmon)	Skin elasticity, hydration, anti-aging, antioxidant, anti-inflammatory	Collagen supplements, functional foods	Xu et al. (2023)
Fish protein hydrolysates	Fish bones, skin, muscle (Tilapia, Tuna, Catfish, Sardine, Atlantic Cod)	Antioxidant, antihypertensive, immunomodulatory, antihyperglycemic	Functional beverages, dietary supplements	Olaniran et al. (2024)
ACE-inhibitory peptides	Fish muscle and bones (Sardine, Salmon, Tilapia)	Antihypertensive, blood pressure regulation	Nutraceuticals, antihypertensive supplements	Abachi et al. (2019)
Antioxidant peptides	Fish skin and scales (Atlantic Salmon, Cod, Mackerel)	Free radical scavenging, metal chelation	Antioxidant supplements, functional foods	Liu et al. (2024)
Anti-inflammatory peptides	Fish scales and skin (Atlantic Salmon, Tilapia)	Inhibition of pro-inflammatory cytokines	Anti-inflammatory nutraceuticals	Abachi et al. (2019)
Neuroprotective peptides	Fish muscle (Cod, Salmon)	Neuroprotection, oxidative stress reduction	Neurohealth supplements	Chataigner et al. (2021)
Anti-diabetic peptides	Fish scales and collagen (Tilapia, Tuna)	Blood sugar regulation, DPP-IV inhibition	Antidiabetic functional foods	Rivero-Pino et al. (2024)
Immunomodulatory peptides	Fish by-products (Shrimp, Cod skin)	Immune enhancement, cytokine modulation	Immune support supplements	Kang et al. (2019)
Bone health peptides	Fish bones (Tilapia, Atlantic Cod)	Bone mineralization, osteoblast activation	Bone health supplements	Pérez et al. (2024)
Wound healing peptides	Fish scales and skin (Cod, Salmon)	Tissue regeneration, collagen synthesis	Wound care products, skin repair supplements	Oslan et al. (2022)
Anti-cancer peptides	Marine fish (Sponges, Cod)	Cytotoxicity against tumor cells	Functional ingredients in cancer prevention	Zhang et al. (2021)
Antimicrobial peptides	Marine fish and mollusks (Abalone, Cod)	Antibacterial and antifungal activity	Natural preservatives, functional foods	Rodrigues et al. (2025)

### Collagen supplements for skin, joint, and bone health

Due to their beneficial influence on skin, joints, and bones, supplements made from marine collagen (found in fish scales, skin, and bones) have gained increasing popularity among nutraceutical consumers. Typically available in the form of hydrolyzed collagen peptides or gelatin, these supplements exhibit higher bioavailability and are more easily digested, thereby effectively supporting the connective tissue system. Moreover, marine collagen’s lower molecular weight, superior solubility, and compatibility with various dietary and religious preferences make it a favourable alternative to mammalian collagen for use in supplements and functional foods (Coppola et al. 2021; Salvatore et al. 2020). Collagen plays a critical role in maintaining the extracellular matrix (ECM), thereby imparting elasticity, hydration, resilience, and structural strength to the dermis (Reilly and Lozano 2021). A natural decline in collagen levels—accelerated by aging, environmental exposure, and lifestyle factors—leads to dryness, reduced flexibility, and the formation of wrinkles and fine lines (Hussein et al. 2025). Numerous studies have consistently demonstrated that oral supplementation with marine-derived collagen peptides can mitigate these signs of aging. For example, in a randomized clinical study involving women aged 35–55, those receiving daily fish collagen peptides for eight weeks exhibited significant improvements in skin hydration, elasticity, and wrinkle reduction compared to the placebo group (Ito et al. 2018). Additional studies revealed that 12 weeks of supplementation increased skin moisture and dermal collagen content while lowering MMP-2 levels, thereby limiting collagen degradation. Acting as bioactive messenger molecules, collagen peptides stimulate fibroblast activity and promote new skin formation, enhancing overall skin health. Consequently, marine collagen peptides are also believed to protect skin cells from UV-induced damage (Zhao et al. 2021).

Beyond skin benefits, marine collagen supplements have demonstrated positive effects on joint health by strengthening cartilage and reducing osteoarthritis-related pain. A portion of hydrolyzed collagen peptides is transported to cartilage tissues, where it stimulates chondrocytes to produce collagen type II and proteoglycans—both vital components of the cartilage ECM (Aigner and Stöve 2003). Steele (Steele 2022) reported that daily intake of 10 g of fish-based collagen peptides for 12 weeks alleviated knee osteoarthritis symptoms, decreasing joint pain and improving mobility. Regular supplementation also reduced joint tenderness and enhanced flexibility, offering a safer, more natural approach than pharmacological treatments (Ashraf et al. 2020). Furthermore, collagen peptides may alleviate inflammation by modulating cytokine activity and oxidative stress in joint tissues (Khatri et al. 2021). Similarly, bone health also benefits from marine collagen intake, as bone tissue is primarily composed of collagen type I, which provides a scaffold for mineral deposition and structural integrity (Viguet-Carrin et al. 2006). Age-related collagen deterioration contributes significantly to bone loss and osteoporosis. Human studies suggest that collagen peptide supplementation promotes bone regeneration and enhances bone strength. In one study, participants who consumed 5 g of collagen peptides combined with calcium and vitamin D daily for 12 months showed increased bone mineral density (BMD) compared to controls (König et al. 2018). This improvement correlated with elevated bone formation markers and reduced resorption markers, indicating positive bone remodeling. Supporting evidence from Zdzieblik et al. (Zdzieblik et al. 2017) further confirmed that collagen peptides stimulate osteoblast activity and reinforce bone matrix structure.

### Safety and Bioavailability

Many studies have shown that marine collagen peptides are safe to use and mildly affect only a small number of people (Felician et al. 2018). Because they have low molecular weight and are easily absorbed, these peptides can bring about detectable increases in plasma levels of hydroxyproline and other collagen metabolites (Skov et al. 2019). Their action in stimulating repair and growth of connective tissues depends on being available to tissues. In addition, marine collagen is usually safer to use than bovine or porcine, since it can’t spread diseases and is more culturally acceptable (Coppola et al. 2021; Subhan et al. 2015).

### Delivery forms and formulation strategies & market trends and consumer demand

Some functional proteins and peptides from sea life have the potential to help protect muscle, control weight and keep the heart healthy. Taking collagen peptides and fish protein hydrolysates can support muscle growth, reduce time to muscle recovery and help reduce sarcopenia in senior adults (Inacio et al. 2024). Proteins in seafood help keep weight in balance because they make you feel full and their active compounds support heart health by controlling the body’s blood pressure, defending against antioxidants and lowering the amount of cholesterol in the blood (Ahhmed and Muguruma 2010). So that consumers can find what works best for them, scientists formulate bioactive ingredients into different types, including powders, capsules, beverages and bars and snacks. Because of these innovations, demand for marine-based nutraceuticals is rising, as more consumers become aware of natural health, request supplements without artificial ingredients and give greater attention to preventive health and staying active (Hosomi et al. 2012). As a result of this trend, experts are focusing on research and developing new products to make use of marine proteins in health care.

## Challenges and limitations of marine-derived proteins and collagen

Even though marine proteins and collagen have potential in nutraceuticals and functional food, a number of key challenges make it hard for these products to be widely used and manufactured on an industrial scale. Issues such as changing materials and complicated regulations affect how well, accurately and easily products are made and sold.

### Variability in raw material quality

A key difficulty in harvesting collagen and functional proteins from fish scales, skins and bones is that the materials differ greatly in quality. The type of fish, its age, where it lives, its preferred food and how the fish is processed strongly influence its chemical structure, collagen levels and protein quality (Abraha et al. 2018). For example, the amino acid profile and heat resistance of fish collagen can differ between cod and salmon, on the one hand and tilapia or catfish on the other (Gaikwad and Kim 2024). At different seasons and after harvesting, the peptide yield and its activity are not always the same. A lack of standardization is a problem here, so getting the same results and trust from customers is very difficult.

### Stability and bioavailability of proteins and collagen

Their bioavailability and tendencies to dissolve make collagen peptides and marine proteins less widely accepted. Although hydrolyzed collagen peptides can be absorbed more easily than native collagen, their digestive stability is reduced, so they may not reach the whole body as well (León-López et al. 2019). Due to environmental factors, peptides subjected to digestion may lose their activity before reaching and affecting the target body tissues. In addition, when collagen peptides are stored or processed, they can change which can influence both their functions and how long they stay usable (Meyer 2019). Investigating how peptides can be stabilized with inventive methods such as encapsulation, nanoemulsions and combined systems is being actively researched, although these techniques increase product development costs (Verma 2023).

### Regulatory considerations and safety

Because of safety, labeling and health claim issues, using and selling marine collagen products can be challenging for manufacturers. Because laws regarding nutraceuticals, functional foods and dietary supplements change from country to country, it is complicated for them to enter the global market (Bagchi 2019). Many authorities such as the FDA in the US, EFSA in Europe and others, insist on thorough assessments of allergens, toxicity and contamination, due to the risk of marine by-products containing heavy metals or pollutants (Emerald and Rosenberg 2022). Further, for collagen to be marketed for its benefits on hair, skin, joints and bones, companies must provide scientific proof that is often tough and time- and money-consuming to gather (König et al. 2018). Regulations and agreements should be simple and matched so that afternoon tea can be innovative and safe.

### Scale-up and cost-effectiveness of extraction processes

One of the biggest barriers to transforming marine by-products into commercially viable nutraceuticals is the challenge of cost and scalability. While laboratory-scale methods such as enzymatic hydrolysis, ultrasound-assisted extraction (UAE), and microwave-assisted extraction (MAE) have demonstrated good yields and strong bioactivity, moving these techniques to industrial-scale operations remains complex and expensive (Panda and Manickam 2019). A major cost driver is the use of enzymes, which are effective in preserving collagen’s molecular integrity but remain costly and sometimes unstable under large-scale processing conditions. Additionally, these processes require controlled energy input, solvents, and multi-step purification stages such as ultrafiltration and chromatography, all of which increase production costs. The reliance on cold chain logistics, sterile environments, and precise process monitoring further add to operational expenses. Raw material variability also complicates scale-up, since fish by-products differ by species, season, diet, and processing methods, leading to inconsistent collagen content and peptide profiles. This lack of uniformity necessitates careful sorting, pre-processing, and quality testing, which further increases costs. Waste management is another hidden expense: before raw material reaches the extraction facility, seafood residues often need to be decontaminated or stabilized to prevent spoilage, adding to logistical and energy burdens (Demirbas 2011). Despite these hurdles, new strategies are being developed to improve economic feasibility. Integrated biorefinery models, where collagen, lipids, chitin, and minerals are co-extracted from the same biomass, can reduce waste and maximize resource use. Process intensification techniques such as continuous-flow bioreactors, enzyme immobilization to enable enzyme reuse, and membrane-integrated extraction systems are being tested to cut costs and improve efficiency. Moreover, advanced “green” technologies like subcritical water extraction (SWE) and deep eutectic solvents (DES) are gaining attention for their ability to lower solvent use and energy requirements (Sonu et al. xxxx). At present, most of these technologies are still in pilot or demonstration phases and have not yet been widely scaled up to full commercial production. The transition from bench to industry will require not only technical optimization but also economic modelling, life-cycle analysis, and regulatory validation to ensure the processes are safe, sustainable, and profitable. Ultimately, achieving cost-effectiveness in large-scale marine collagen production will depend on combining innovative extraction technologies with integrated waste-to-value strategies that align with circular bioeconomy principles.

## Future perspectives and research directions

Because more individuals are interested in natural and effective nutraceuticals and new biotechnologies are being developed, interest in marine-derived proteins and collagen is increasing quickly. Working on today’s problems and searching for new solutions helps develop the therapeutic and commercial usefulness of marine biomolecules.

### Innovations in extraction and processing technologies

Future studies will likely find innovative ways to produce marine proteins and collagen that are economical, sustainable and maintain product quality. New processes such as using enzymes, combining membranes with bioreactors and having continuous flow systems show promise for raising yield and keeping costs down (Padhan et al. 2023). In particular, using immobilized enzymes makes it possible to use them again, making collagen peptide hydrolysis more stable and easier to control (Bezerra et al. 2015). Furthermore, deep eutectic solvents (DES) and subcritical water extraction (SWE) are preferred now since they are environmentally friendly, still allow bioactivity and lower waste of solvents (Zhang et al. 2020; Cheng et al. 2021). Optimizing the process by combining UAE and MAE techniques with ultrafiltration and chromatographic purification is being carried out to maintain the quality of extracted peptides in ongoing industrial use (Mohamad Razali et al 2023; Shakoor et al. 2023). These advances should increase how well marine peptides can be extracted, while also making them more stable and easier for the body to use, overcoming issues seen with regular methods.

### Novel applications in emerging nutraceutical products

Besides collagen supplements and joint support products, marine proteins and collagen peptides are starting to be added to a variety of new nutraceuticals designed to promote better health. Scientists have started working on targeted delivery methods, like nanoencapsulation and liposomal carriers which increase peptide stability and help control when and where in the body they are active (Pinto Reis et al. 2006; Zhang et al. 2022). Coupling marine peptides with other health-promoting ingredients benefits anti-aging, heart care and good metabolic health. People are becoming more interested in making nutraceuticals using marine peptides that match an individual's genes and metabolism to enhance overall health. New technologies in multi-omics and bioinformatics is leading to innovative uses of marine peptides in various sectors.

### Integration with other marine biomolecules for synergistic effects

One useful development would be to join marine collagen and proteins with other marine-derived compounds like polysaccharides (such as fucoidan and chitosan), omega-3 fatty acids and polyphenols. It has been proposed that by including a range of anti-inflammatory, antioxidant and immunomodulatory effects in the formulation, these products could amplify how well they work (Sivaraman and Shanthi 2021; Chotphruethipong et al. 2021). Taking collagen peptides together with fucoidan was found to provide better skin benefits and anti-aging results compared to either ingredient taken alone (Charoensiddhi et al. 2023). In future, we can get better cardiovascular, bone and metabolic health effects by combining marine extracts such as polysaccharides, lipids and peptides (Hamed et al. 2015). Still, there are numerous problems connected with these products, including stability, compatibility and regulatory rules, but the chance for them to work together in helpful ways is a good reason to continue investigating. Besides, marine biorefineries are being built to separate waste from seafood processing into separate benefits. This helps make the marine nutraceutical industry more sustainable.

### Future directions

**Fig. 6:**
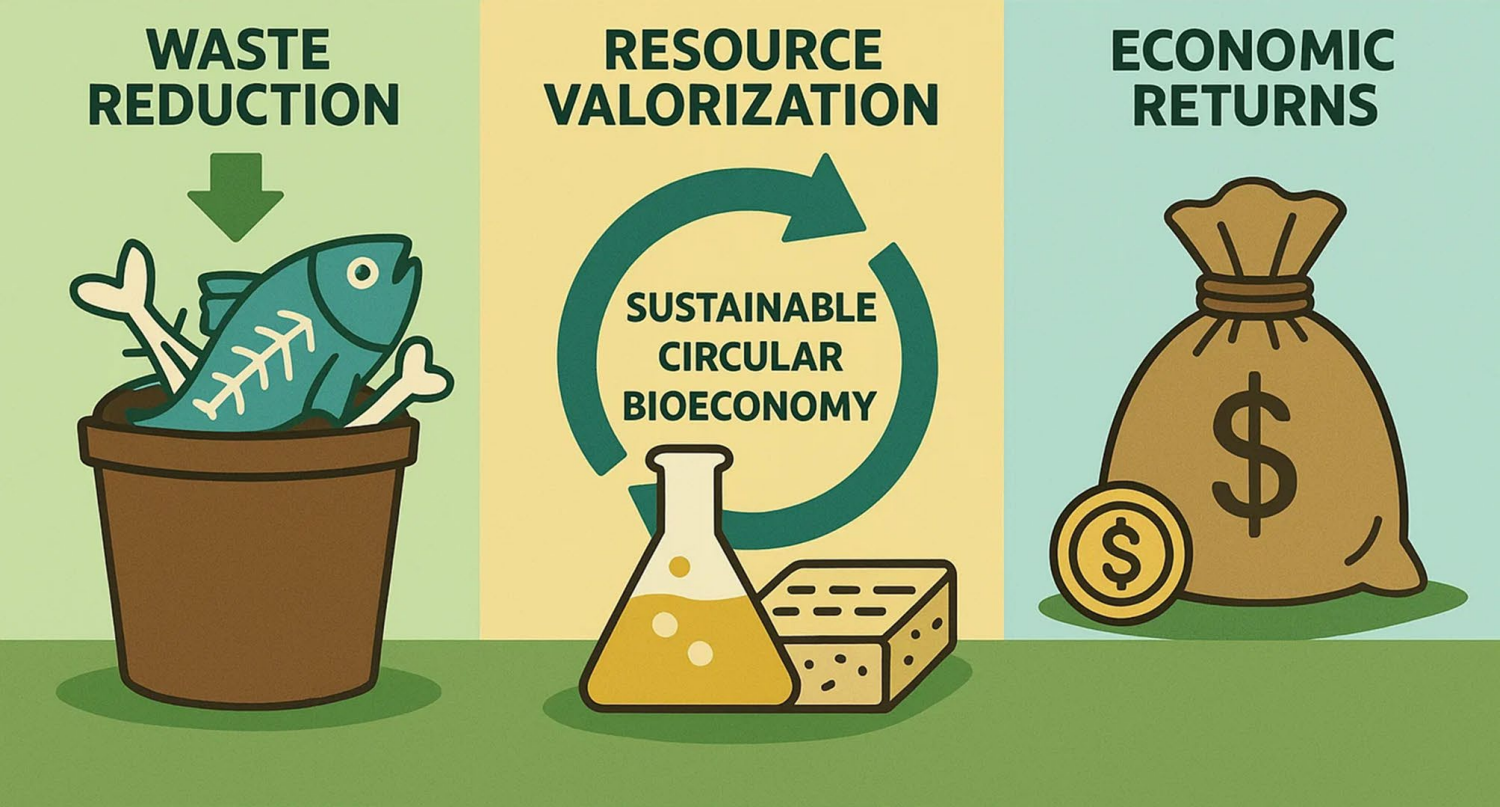
Environmental and economic benefits of utilizing fish scale and bone waste

The potential of fish scales and bones as sustainable sources of collagen and bioactive peptides is clear; however, moving promising laboratory findings into robust commercial nutraceuticals requires addressing three major bottlenecks: scalability and process economics, regulatory and safety pathways, and product stability, bioavailability, and sensory acceptability (Fig. 6). Overcoming these barriers is essential for translating research outputs into viable nutraceutical products. In terms of scalability and process economics, many advanced extraction methods such as ultrasound-assisted extraction, microwave-assisted extraction, enzymatic hydrolysis, and subcritical water extraction have shown high efficiency and improved bioactivity at laboratory scale. However, cost and technical barriers emerge when scaling up these processes. Challenges include the high cost of enzymes, energy-intensive operations, solvent recovery, and variability in raw feedstock availability. Promising solutions include process intensification strategies such as continuous biomanufacturing, enzyme immobilization to allow reuse and reduce costs, and the adoption of integrated biorefinery models where collagen, lipids, and minerals are co-processed to enhance overall economic and environmental sustainability.

From a regulatory and safety perspective, nutraceuticals derived from fish by-products face a heterogeneous global landscape. Requirements for claims, allergen labelling, contaminant limits, and quality standards vary widely across jurisdictions, making global commercialization difficult. In addition, potential contamination by heavy metals, persistent organic pollutants, and microbial hazards in marine by-products demands validated decontamination procedures, traceable supply chains, and rigorous quality control. Early engagement with regulatory authorities and implementation of Good Manufacturing Practice (GMP) standards are essential steps for ensuring both compliance and consumer trust. Another critical challenge involves stability, bioavailability, and sensory acceptance. The requirements for claims, allergen labelling, contaminant limits and quality standards vary widely across jurisdictions, making global commercialization difficult. Bioactive peptides derived from fish by-products often exhibit strong in vitro activity but suffer from poor oral stability and limited systemic absorption due to enzymatic degradation. Furthermore, hydrolysates frequently carry a bitter taste that can reduce consumer acceptance. Advances in nanotechnology-enabled delivery systems offer promising solutions. Nanoencapsulation approaches, including nanoliposomes, polymeric nanoparticles, and nanoemulsions, have been shown to improve the stability, bioavailability, and controlled release of bioactive peptides. These technologies can also mask undesirable tastes and enhance sensory attributes. For example, recent work by Nemati et al. (Nemati et al. 2024) highlights the potential of nanoliposomes and related carriers to protect fish protein hydrolysates, reduce bitterness, and enhance consumer acceptance.

To implement these solutions effectively, research groups and industries should prioritize standardization of raw-material sourcing and quality control, pilot continuous processing with immobilized enzyme systems, screening of nanocarrier formulations for stability and sensory improvements, and early regulatory mapping supported by toxicological and pharmacokinetic evaluations. Integrating process intensification strategies with advanced delivery platforms such as nanotechnology provides a practical roadmap to overcome the identified barriers. Ultimately, combining sustainable extraction processes with smart delivery systems can accelerate the translation of fish by-product–derived nutraceuticals into large-scale, consumer-acceptable, and regulatory-compliant products.

## Conclusion

Fish scales and bones are in great supply, have a light impact and are not commonly used, making them promising for making high-value protein and collagen products for nutraceuticals. Thanks to abundant type I collagen and bioactive peptides in their composition, they promote good health by fighting free radicals, lowering inflammation, lowering blood pressure, helping the skin, aiding healthy joints and boosting bones. Better green methods for extracting and purifying collagen have improved its quality, use and impact on the environment, as well as reducing what is thrown away from the seafood industry. Although there are promising uses, better raw material standards, consistent bioavailability and product stability, regulations and production are still obstacles in the field. Development and experimentation with advanced ways to obtain and blend fish scale and bone-based proteins and collagen, together with more marine biomolecules, will be important to both use and market these nutraceuticals effectively for healthy living and cleaner environments.

## Data Availability

All data are available in manuscript.
